# Supplementary material for: KSNP: a fast de Bruijn graph-based haplotyping tool approaching data-in time cost
Source: Nat Commun. 2024 Apr 11;15:3126. doi: 10.1038/s41467-024-47562-4 (PMC11009271; doi:10.1038/s41467-024-47562-4)
Supplement: Supplementary file 1 — Supplementary Information [file 41467_2024_47562_MOESM1_ESM.pdf]

KSNP: a fast de Bruijn graph-based haplotyping tool approaching data-in time cost

Table of contents 1

Supplementary Note 1 Algorithm for graph pruning in KSNP 2

Supplementary Note 2 How KSNP handles complex bubbles.................................4

Supplementary Note 3 Command lines for replicating the analyses......................5

Supplementary Figure 1..7

Supplementary Table 1 8

Supplementary Table 2 9

Supplementary Table 3 10

Supplementary Table 4 11

Supplementary Table 5 12

Supplementary Table 6 13

Supplementary Table 7 14

Supplementary Table 8 16

Supplementary Table 9 17

Supplementary Table 10 18

Supplementary Note 1 Algorithm for graph pruning in KSNP

KSNP builds a DBG of k by collecting k-mers on each row in read-variant matrix, which is usually used to describe read partitioning problem for solving MEC. The columns of matrix are heterozygous SNPs. Each row contains aligned alleles in a read, with 0 for reference allele, 1 for alternative allele and character – for gap (deletion in alignment). k-mers are substrings of length k in a row without gaps. A k-mer includes two parts of information, k alleles that are encoded as a k-bit integer w and the SNP index p of the first allele. In construction of graph, same k-mers are collapsed into one node with the occurrence number as weight. Thus, a node can be uniquely represented as V_p^w. The read spanning two adjacent k-mers adds an edge between their nodes. Similarly, repetitive edges are used to increase the edge weight. E_p^w is used to encode an edge between V_p^{w1} and V_{p+1}^{w2}, and w is a (k+1)-bit word combined from $w1$ shifting one bit left and the most insignificant bit of $w2$. And W (E_p^w) is used to denote the weight of an edge. Because of the symmetry of diploid genomes, the complement of nodes and edges are added to graph at the same time.

With the k-mer size of K and the heterozygous SNP number of N, we describe in detail the major steps of graph pruning in Algorithm 1.

Algorithm 1: Resolving DBG

1 **for** $p \leftarrow 0$ **to** $N - K + 1$ **do** /* Step 1: Trimming light-weight edges */
2 $M \leftarrow$ the maximum edge weight in $E_p^0 \ldots E_p^{2^{K+1}-1}$
3 **for** $w \leftarrow 0$ **to** $2^{K+1} - 1$ **do**
// C is a certain confidence threshold
4 **if** $M \geq C$ ***and*** $2 * W(E_p^w) < M$ **then** remove E_p^w
5 **if** $M < C$ ***and*** $5 * W(E_p^w) < M$ **then** remove E_p^w
6 **for** $p \leftarrow 0$ **to** $N - K$ **do** /* Step 2: Removing short branches */
7 **for** $w \leftarrow 0$ **to** $2^K - 1$ **do**
8 **if** *outdegree*(V_p^w) $= 2$ **then**
9 $P^0 \leftarrow$ path starting from E_p^{2w}
10 $P^1 \leftarrow$ path starting from E_p^{2w+1}
11 **if** ***not*** *both* P^0 *and* P^1 *are linear path* **then continue**
12 **if** $|P^0| < 3 * |P^1|$ **then** remove all edges on P^1
13 **if** $|P^1| < 3 * |P^0|$ **then** remove all edges on P^2
14 Lines from 6 to 13 is topologically reversed executed
15 *bubbles* $\leftarrow \emptyset$ /* Step 3: Solving bubbles */
16 **foreach** *node* S *in graph* **do**
17 **if** *outdegree*(S) $\neq 2$ **then continue**
18 $T \leftarrow$ the terminate node of two extend outgoing edges
19 $R \leftarrow$ read set included from S to T
20 $B \leftarrow$ path number between S and T
21 Add $[S, T, R, B]$ to *bubbles*
22 Merging bubbles with intersections on read sets
23 **foreach** $[S, T, R, B]$ *in bubbles* **do**
24 $L \leftarrow \emptyset$ // the best path to keep
25 **if** $B < 512$ **then**
26 $M \leftarrow \infty$
27 **foreach** *path* P *between* S *and* T **do**
// H(P) is haplotype of P; MEC is calculated using R
28 $m = MEC(H(P), R)$
29 **if** $m < M$ **then** $M \leftarrow m, L \leftarrow P$
30 **else**
31 $L \leftarrow$ the path with maximum sum weight
32 **for** $t \leftarrow 1$ **to** 512 **do**
33 $P \leftarrow L$ replaced with some other edges following a template
34 **if** $MEC(H(P), R) < MEC(H(L), R)$ **then** $L \leftarrow P$
35 **break** until MEC stops change
36 Remove all paths in bubble except to L
37 Completing remanning branches in step 2 into bubbles, and solve them using step 3.
38 **return** haplotype blocks read from linear paths

Supplementary Note 2 How KSNP handles complex bubbles

In simple bubble with limited number of haplotype paths, we can enumerate each possible solution and decided the best one through calculating MEC score. Bus it is not allowed in complex cases because the complexity could be exponential. To efficiently find the optimal or near-optimal path, we adopt a heuristic algorithm to solve the problem. At first, an initial path with the maximum weight sum is chosen by dynamic programming. It is better than a random path but still contains many switch errors. We iteratively update the path by replacing some edges on the condition of improving MEC score. This process is similar to the MAX-CUT algorithm in HapCUT2, where each cut flips phasing of a set of SNPs and gradually reduces the errors on the updating haplotypes. Instead of computational cut operations, KSNP uses a list of fixed templates to correct local errors which frequently happen in the DBG. First, from each node with two outgoing edges on the updating path, KSNP switches to the other edge and go along the complementary paths, for example from '00→00→00→00' to '00→01→11→11', and performs update if this operation leads to a reduction of MEC score. Then, KSNP tries to replace two adjacent edges to correct mismatches like 'FFMFF'. Scaling to dense errors, KSNP will consider more adjacent edges and replacement combinations, which are called 'cut templates' to decide which edges should be replaced. Templates not improving MEC efficiently will be discarded and not used in further updating. The local updates are real-time and marked in a bit-array, so subsequent MEC score is calculated without explicitly modifying the graph. In the end, the template length does not increase infinitely, the whole process stops until the MEC score does not change in 5 rounds or the total number of iterations is up to 512.

Supplementary Note 3 Command lines for replicating the analyses

Mapping reads

Mapping ONT, CLR reads with **minimap2** (version 2.17, https://github.com/lh3/minimap2). Sort and index BAM file with **samtools** (versioni 1.9, https://github.com/samtools/samtools/releases).

```
minimap2 -ax map-ont -t16 ref.fasta reads.fastq.gz| samtools view -hb -F 0x904 > unsorted.bam
minimap2 -ax map-pb -t16 ref.fasta reads.fastq.gz | samtools view -hb -F 0x904 > unsorted.bam
samtools sort -@16 -o sorted.bam unsorted.bam
samtools index sorted.bam
```

The CLR mapping with **BLASR** (version 5.1, https://anaconda.org/bioconda/blasr).

```
blasr seqs.fastq.gz ref.fasta --nproc 32 --bestn 1 --bam -out unsorted.bam
```

Subsampling with samtools

```
samtools view -s 0.FRAC -b input.bam | samtools view -hb > output.bam
```

Variant Calling

Variant calling by **Longshot** (versoin 0.4.1, https://github.com/pjedge/longshot).

```
longshot --no_haps --max_cov 500 --bam aln.bam --ref ref.fa --out variant.vcf
```

Variant calling for ONT reads by **PEPPER-Margin-DeepVariant** (PMD, version r0.7, https://github.com/kishwarshafin/pepper).

```
singularity exec -B /data_path:/base --bind /usr/lib/locale/ \
    pepper_deepvariant_r0.7.sif \
    run_pepper_margin_deepvariant call_variant \
    -b /base/aln.bam \
    -f /base/ref.fa \
    -o /base/output/ \
    -t 24 \
    --ont_r9_guppy5_sup
```

Or run **PMD** with docker.

```
sudo docker run \
    -v /data_path:/base \
    kishwars/pepper_deepvariant:r0.7 \
    run_pepper_margin_deepvariant call_variant \
    -b /base/aln.bam \
    -f /base/ref.fa \
```

```
  -o /base/output/ \
  -t 24 \
  --ont_r9_guppy5_sup
```

The raw variant files should be properly filtered before performing the phasing.

Phasing

Whatshap version 1.7 from https://github.com/whatshap/whatshap/.

```
whatshap phase --ignore-read-groups --reference ref.fa --output phased.vcf variant.vcf
aln.bam
```

Longshot

```
longshot --potential_variants variant.vcf --max_cov 500 --bam aln.bam --ref ref.fa
—out phased.vcf
```

HapCUT2 version 1.3.1 from https://github.com/vibansal/HapCUT2.

```
extractHAIRS --bam aln.bam --VCF variant.vcf --ref ref.fa --ont 1 --out inter.frag
extractHAIRS --bam aln.bam --VCF variant.vcf --ref ref.fa --pacbio 1 --out inter.frag
HAPCUT2 --fragments inter.frag --VCF variant.vcf --output out_prefix
```

Margin version 2.3.1 from https://github.com/UCSC-nanopore-cgl/margin.

```
margin phase aln.bam ref.fa variant.vcf \
  params/phase/allParams.phase_vcf.ont.json \ # allParams.phase_vcf.pb-hifi.json if CLR data
  -o out_prefix -t 16 --skipHaplotypeBAM
```

KSNP version 1.0 from https://github.com/zhouqiansolab/KSNP.

```
ksnp -b aln.bam -r ref.fa -v variant.vcf -o phased.vcf
```

Supplementary Figure

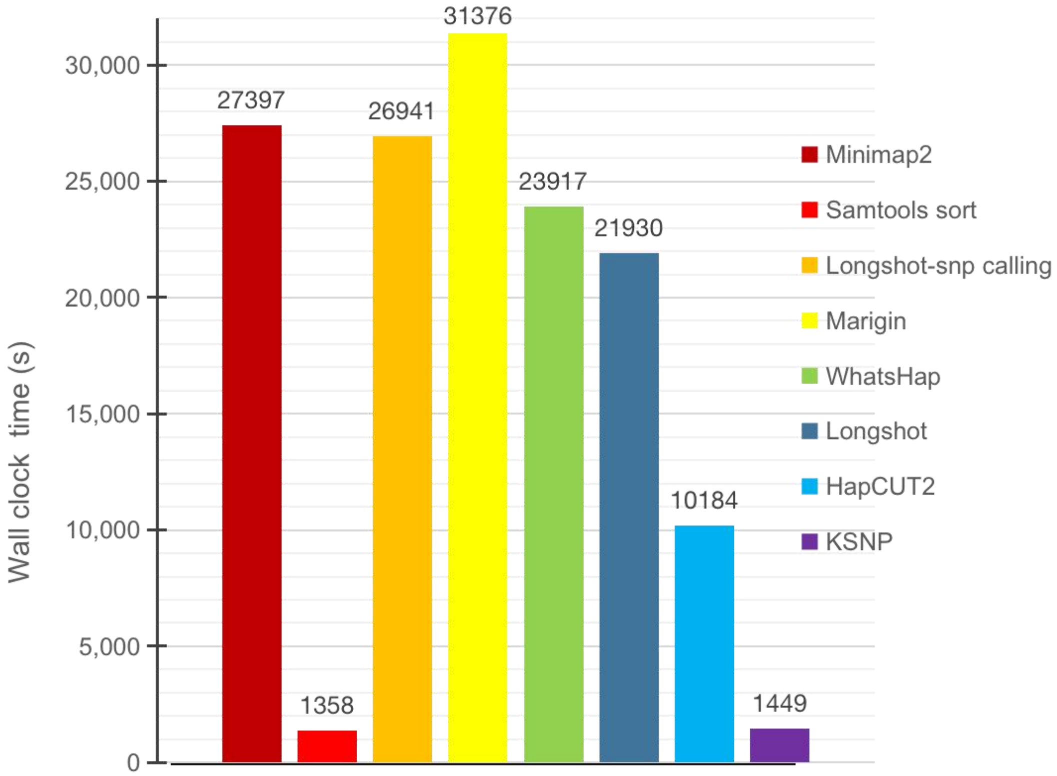

Supplementary Figure 1 The wall clock time for each processing step of the HG01109 data For read mapping and sorting, we used 4 threads for Minimap2 and SAMtools. SNP calling was performed with Longshot (--no_haps) using 1 thread for it is single-threaded. For phasing, we used 4 threads for Margin and 1 thread for other tools as they are single-threaded. Minimap2, Longshot for SNP calling, Margin, WhatsHap, Longshot for phasing, HapCUT2, and KSNP consumed 7.6, 7.5, 8.7, 6.6, and 6.1, 2.8 and 0.4 hours, respectively.

Supplementary Table 1 Data metric used in experiments

Sample	Data type	Data size (Gb)	Read N50 (kb)	SNP per read [a]	Ground Truth	Reference genome
HG001	CLR	176.9	6.1	6.6	GIAB v3.3.2	GRCh37
	ONT	147.4	19.5	15.3		
HG002	CLR	148	11.0	9.6	GIAB v4.2.1	GRCh37
	HiFi	110.5	14.7	15.91		
	ONT	152.2	50.3	24.9		
HG005	CLR	176.4	16.3	12.6	GIAB	GRCh37
	ONT	174.5	49.3	30.1		
HG01109	CLR	122.4	32.5	23.3	Parent read	GRCh37
A.thaliana F1	CLR	7.1	17.6	36.01	Parent read	TAIR10

[a] The variant callers were Longshot for CLR and HiFi data, and Pepper-Margin-DeepVariant for ONT data. Only high-quality heterozygous SNPs and reads containing at least 2 SNPs were involved in this statistic.

Supplementary Table 2 Evaluation of different K values on CLR reads with BLASR as read mapper

Dataset	K	Switch error rate (%)	Hamming error rate (%)	Haplotye N50 (kb)	Recall rate(%)
HG001 (~50x)	2	0.68	1.82	246	91.59
	3	0.66	1.63	243	90.98
	4	0.66	1.54	221	90.05
	5	0.68	1.44	191	88.52
HG002 (~50x)	2	1.25	2.12	321	89.91
	3	1.24	1.94	320	89.39
	4	1.24	1.85	296	88.76
	5	1.27	1.76	268	87.87
HG005 (~50x)	2	1.49	3.98	514	92.80
	3	1.47	3.55	493	92.39
	4	1.46	2.89	448	91.83
	5	1.46	2.60	420	91.21
HG01109 (~45x)	2	0.07	5.94	5,333	89.66
	3	0.07	4.85	4,954	89.63
	4	0.07	4.99	4,302	89.56
	5	0.06	3.72	3,623	89.49
A.thaliana F1(~45x)	2	0.01	2.56	4,001	85.98
	3	0.01	2.06	4,001	85.98
	4	0.01	1.65	4,001	85.98
	5	0.02	2.03	4,001	85.97

Supplementary Table 3 Evaluation of different K values on CLR reads with minimap2 as read mapper

Dataset	K	Switch error rate (%)	Hamming error rate (%)	Haplotye N50 (kb)	Recall rate(%)
HG001 (~50x)	2	0.64	1.67	243	91.34
	3	0.63	1.49	240	90.72
	4	0.63	1.40	218	89.79
	5	0.66	1.45	187	88.23
HG002 (~45x)	2	1.23	1.93	323	90.19
	3	1.22	1.77	323	89.68
	4	1.22	1.62	301	89.04
	5	1.24	1.67	272	88.15
HG005 (~50x)	2	1.48	3.95	532	92.82
	3	1.46	3.39	506	92.41
	4	1.45	2.90	466	91.86
	5	1.45	2.82	425	91.26
HG01109 (~40x)	2	0.08	5.14	5,758	89.98
	3	0.07	4.53	5,293	89.95
	4	0.08	3.05	4,370	89.87
	5	0.08	2.63	3,600	89.80
A.thaliana F1 (~45x)	2	0.02	1.82	5,908	87.68
	3	0.02	1.84	5,908	87.68
	4	0.02	2.13	5,908	87.68
	5	0.02	1.23	5,908	87.68

Supplementary Table 4 Evaluation of different K values on ONT reads with Longshot as variant caller

Dataset	K	Switch error rate (%)	Hamming error rate (%)	Haplotye N50 (kb)	Recall rate(%)
HG001 (~50x)	2	0.68	16.43	7,361	92.93
	3	0.68	13.70	5,678	92.89
	4	0.68	10.99	4,053	92.79
	5	0.68	8.94	3,192	92.64
HG002 (~50x)	2	1.28	24.62	25,440	90.99
	3	1.25	24.41	24,728	91.01
	4	1.24	23.01	19,853	91.01
	5	1.29	19.93	15,055	90.95
HG005 (~50x)	2	1.53	31.50	27,028	91.45
	3	1.50	30.48	24,494	91.47
	4	1.49	27.97	18,768	91.48
	5	1.49	23.43	11,057	91.45

Supplementary Table 5 Evaluation of different K values on ONT reads with PEPPER-Margin-Deepvariant as variant caller

Dataset	K	Switch error rate (%)	Hamming error rate (%)	Haplotye N50 (kb)	Recall rate(%)
HG001 (~50x)	2	0.67	5.37	4,921	92.36
	3	0.68	5.38	4,370	92.32
	4	0.68	3.86	3,720	92.22
	5	0.68	3.50	3,011	92.07
HG002 (~50x)	2	1.24	6.18	13,103	90.03
	3	1.24	7.40	13,077	90.03
	4	1.24	6.50	11,964	90.01
	5	1.24	6.22	9,793	89.96
HG005 (~50x)	2	1.46	7.03	8,500	90.42
	3	1.46	6.56	7,279	90.40
	4	1.46	6.19	5,971	90.35
	5	1.47	4.48	4,576	90.23

Supplementary Table 6 Real time consumption of each step of KSNP

Dataset	Read BAM&VCF (s)	Re-alignment (s)	DBG (s)	Output (s)	Total(s) [a]
HG001 CLR 50x	1672.1	176.2	69.2	10.4	2004.0
HG002 CLR 50x	1458.2	158.0	54.4	10.3	1739.7
HG005 CLR 50x	1892.9	221.7	72.8	9.7	2268.1
HG01109 CLR 40x	1134.5	158.8	106.1	12.9	1449.2
HG001 ONT 50x	1992.9	117.9	141.5	7.8	2307.2
HG002 ONT 50x	2123.1	139.6	219.6	7.7	2536.2
HG005 ONT 50x	2442.7	154.3	221.3	7.2	2865.7
chr1[a]	136.2	13.7	5.9	0.7	162.9
chr2	146.2	14.9	4.7	0.8	172.8
chr3	118.6	12.3	3.7	0.7	140.9
chr4	118.1	12.4	3.8	0.7	140.2
chr5	109.3	11.7	3.6	0.7	130.1
chr6	104.2	11.4	3.4	0.6	124.2
chr7	94.0	9.9	3.1	0.6	112.4
chr8	89.5	9.6	3.1	0.6	107.2
chr9	73.2	8.0	2.7	0.5	88.2
chr10	83.2	8.7	3.5	0.6	100.2
chr11	80.4	8.6	2.7	0.5	96.1
chr12	80.1	8.4	2.6	0.5	95.7
chr13	61.7	6.6	1.8	0.4	73.8
chr14	54.4	5.8	2.2	0.3	65.8
chr15	50.4	5.0	2.0	0.3	60.5
chr16	50.9	5.4	2.2	0.3	61.8
chr17	50.1	4.6	1.4	0.3	59.3
chr18	49.1	5.3	1.6	0.3	58.8
chr19	35.1	3.8	1.5	0.3	42.6
chr20	40.7	4.3	1.4	0.2	49.0
chr21	26.0	3.2	4.5	0.4	35.6
chr22	20.5	2.4	1.5	0.2	25.9

[a] Time consumption of each chromosome is based on HG001-CLR-50x dataset.

Supplementary Table 7 Benchmarking results for CLR data

Dataset	Cov	Tool[a]	SE[c] (%)	HE[c] (%)	Hap N50 (kb)	Recall (%)	CPU time (s)	Wall time[c] (s)	RAM[c] (MB)
HG 001	25×	Longshot	0.66	1.37	166	82.70	7,082	7,572	2,253
		WhatsHap	0.71	2.04	168	82.78	13,835	14,437	1,331
		HapCUT2	0.72	2.34	177	82.85	2,923	3,139	523
		Margin	0.66	0.75	98	72.01	72,766	9,210	2,048
		KSNP[b]	0.72	2.13	170	82.74	923	999	395
	50×	Longshot	0.63	1.14	239	91.56	13,485	14,186	2,970
		WhatsHap	0.67	1.57	241	91.61	20,714	22,050	1,843
		HapCUT2	0.67	1.84	251	91.66	6,100	6,478	969
		Margin	0.63	0.89	171	90.30	146,551	19,283	2,867
		KSNP	0.68	1.82	246	91.59	1,881	2,004	510
HG 002	25×	Longshot	1.23	1.60	227	80.67	7,186	7,591	2,048
		WhatsHap	1.27	2.25	231	80.77	12,169	12,848	1,024
		HapCUT2	1.28	2.45	238	80.82	2,908	3,113	524
		Margin	1.20	1.18	136	68.45	65,616	8,748	1,946
		KSNP	1.27	2.42	235	80.76	775	862	376
	50×	Longshot	1.22	1.53	315	89.86	13,537	14,267	2,662
		WhatsHap	1.25	2.03	317	89.91	19,141	19,963	1,536
		HapCUT2	1.25	2.25	324	89.95	6,042	6,453	802
		Margin	1.21	1.38	244	89.17	128,479	16,471	2,458
		KSNP	1.25	2.12	321	89.91	1,607	1,740	476
HG 005	25×	Longshot	1.52	4.19	427	88.58	9,552	10,073	2,253
		WhatsHap	1.64	7.81	491	88.75	14,778	15,394	1,229
		HapCUT2	1.63	7.89	516	88.79	4,301	4,595	543
		Margin	1.44	2.15	317	81.97	82,529	10,316	1,843
		KSNP	1.65	7.65	493	88.74	1,003	1,118	408
	50×	Longshot	1.45	2.77	490	92.75	17,968	18,865	2,765
		WhatsHap	1.49	4.01	507	92.80	22,968	24,093	1,434
		HapCUT2	1.49	4.43	528	92.84	8,202	8,659	946
		Margin	1.44	2.40	315	91.23	145,455	18,648	2,253
		KSNP	1.49	3.98	514	92.80	2,088	2,268	513
HG 011 09	20×	Longshot	0.08	6.58	3,095	78.91	10,410	11,005	2,048
		WhatsHap	0.15	10.03	3,960	79.02	13,615	14,212	997
		HapCUT2	0.17	12.03	4,079	79.03	4,494	4,792	536
		Margin	0.16	10.93	2,823	80.75	67,277	8,737	1,536

		KSNP	0.18	11.26	3,920	79.00	687	774	397
		Longshot	0.04	4.92	5,080	89.61	20,794	21,930	2,662
		WhatsHap	0.06	5.42	5,323	89.66	22,919	23,917	1,331
	40×	HapCUT2	0.07	6.11	5,717	89.67	9,615	10,184	849
		Margin	0.03	2.46	3,804	89.47	125,506	16,513	2,355
		KSNP	0.07	5.94	5,333	89.66	1,304	1,449	512
		Longshot	0.01	5.28	3,772	78.05	2,744	2,755	621
		WhatsHap	0.02	5.37	3,772	78.09	2,039	2,083	577
	20×	HapCUT2	0.02	6.49	3,772	78.09	1,384	1,386	270
		Margin	0.01	2.47	1,289	66.29	4,624	585	1,331
A.thaliana		KSNP	0.03	5.41	3,772	78.09	68	72	125
		Longshot	0.01	2.59	4,001	85.97	3,570	3,586	911
		WhatsHap	0.01	2.12	4,001	85.98	2,610	2,655	615
	45×	HapCUT2	0.01	2.43	4,001	85.99	1,753	1,759	392
		Margin	0.01	2.07	1,228	85.37	9,619	1,202	2,150
		KSNP	0.01	2.56	4,001	85.98	100	100	152

[a] Longshot, WhatsHap, HapCUT2 and KSNP were performed with one thread in the experiments, while Margin utilized eight threads.

[b] The k value in KSNP was set to 2 by default.

[c] SE, switch error rate; HE, hamming error rate; Wall time, Wall clock time; RAM, Peak RAM.

Supplementary Table 8 Benchmarking results for ONT data

Data set	Cov.	Tool[a]	SE[c] (%)	HE[c] (%)	Hap N50 (kb)	Recall (%)	CPU time (s)	Wall time[c] (s)	RAM[c] (MB)
HG001	25×	Longshot	0.68	2.78	2,077	85.45	10,712	11,429	2,048
		WhatsHap	0.70	3.63	2,130	85.91	13,424	14,237	1,024
		HapCUT2	0.70	3.87	2,229	85.91	4,865	5,246	523
		Margin	0.68	0.68	639	84.34	74,345	5,343	1,536
		KSNP[b]	0.70	3.52	2,140	85.90	1,084	1,275	373
	50×	Longshot	0.66	3.27	4,576	91.64	21,249	22,540	2,765
		WhatsHap	0.67	5.14	4,902	92.36	19,989	21,018	1,434
		HapCUT2	0.67	4.64	5,208	92.36	10,284	10,930	867
		Margin	0.66	1.13	1,504	91.97	151,309	19,650	2,253
		KSNP	0.67	5.37	4,921	92.36	2,091	2,307	486
HG002	25×	Longshot	1.24	7.92	8,812	83.55	15,328	16,335	2,048
		WhatsHap	1.26	8.88	9,783	84.07	15,291	16,030	928
		HapCUT2	1.26	8.95	9,900	84.07	7,262	7,764	525
		Margin	1.25	2.83	3,189	83.07	87,614	6,185	1,536
		KSNP	1.26	8.99	9,591	84.07	1,249	1,463	368
	50×	Longshot	1.23	5.38	13,074	89.21	30,954	32,919	2,662
		WhatsHap	1.24	5.76	13,103	90.03	23,909	25,274	1,229
		HapCUT2	1.24	6.08	13,103	90.03	15,645	16,652	842
		Margin	1.23	3.65	7,590	89.73	169,673	22,623	1,741
		KSNP	1.24	6.18	13,103	90.03	2,265	2,536	476
HG005	25×	Longshot	1.48	7.35	4,415	84.62	16,232	17,271	2,150
		WhatsHap	1.49	8.51	4,735	85.22	14,378	15,252	923
		HapCUT2	1.49	9.47	4,985	85.22	7,847	8,385	522
		Margin	1.47	3.03	2,013	84.32	94,086	6,512	1,843
		KSNP	1.49	9.21	4,746	85.22	1,361	1,543	383
	50×	Longshot	1.45	5.50	7,400	89.50	32,509	34,514	2,765
		WhatsHap	1.46	7.75	8,257	90.42	22,990	24,146	1,229
		HapCUT2	1.46	6.46	8,621	90.42	16,633	17,724	973
		Margin	1.45	3.13	3,757	90.11	192,759	24,712	1,741
		KSNP	1.46	6.72	8,388	90.42	2,558	2,866	505

[a] Longshot, WhatsHap, HapCUT2 and KSNP were performed with one thread in the experiments, while Margin utilized eight threads.

[b] The k value in KSNP was set to 2 by default.

[c] SE, switch error rate; HE, hamming error rate; Wall time, Wall clock time; RAM, Peak RAM.

Supplementary Table 9 Phasing with SNPs and reads from different sequencing platforms

	Raw SNPs from long-reads			Raw SNPs from Illumina reads		
**KSNP**	HiFi	CLR	ONT	HiFi	CLR	ONT
Switch error (%)	1.27	1.25	1.24	1.14	1.16	1.14
Hamming error (%)	1.46	2.12	6.18	1.93	2.02	10.68
Haplotye N50 (kb)	422	321	13,103	426	329	14,597
Recall rate (%)	93.68	89.91	90.03	94.13	93.55	95.44
**Longshot**						
Switch error (%)	1.26	1.22	1.23	1.11	1.12	1.11
Hamming error (%)	1.21	1.53	5.38	1.21	1.46	4.1
Haplotye N50 (kb)	416	315	13,074	414	318	13,412
Recall rate (%)	93.67	89.86	89.21	93.54	93.26	94.44
**WhatsHap**						
Switch error (%)	1.27	1.25	1.24	1.14	1.16	1.14
Hamming error (%)	1.48	2.03	5.76	1.86	2.03	8.95
Haplotye N50 (kb)	417	317	13,103	422	322	14,382
Recall rate (%)	93.68	89.91	90.03	94.13	93.59	95.43
**HapCUT2**						
Switch error (%)	1.27	1.25	1.24	1.14	1.16	1.13
Hamming error (%)	1.41	2.25	6.08	1.71	2.12	8.03
Haplotye N50 (kb)	417	324	13,103	422	331	14,881
Recall rate (%)	93.68	89.95	90.03	94.13	93.63	95.45

Supplementary Table 10 Data availability

Sample	Data type	Data source
HG001	CLR reads	https://ftp-trace.ncbi.nlm.nih.gov/giab/ftp/data/NA12878/NA12878_PacBio_MtSinai/
HG001	ONT reads	https://s3-us-west-2.amazonaws.com/human-pangenomics/index.html?prefix=NHGRI_UCSC_panel/HG001/nanopore/Guppy_4.2.2/
HG001	Ground truth	https://ftp-trace.ncbi.nlm.nih.gov/giab/ftp/release/NA12878_HG001/NISTv3.3.2/GRCh37/
HG002	CLR reads	https://ftp-trace.ncbi.nlm.nih.gov/giab/ftp/data/AshkenazimTrio/HG002_NA24385_son/PacBio_MtSinai_NIST/PacBio_fasta/
HG002	ONT reads	https://s3-us-west-2.amazonaws.com/human-pangenomics/index.html?prefix=NHGRI_UCSC_panel/HG002/nanopore/Guppy_4.2.2/GM24385_*_Guppy_4.2.2_prom.fastq.gz
HG002	HiFi reads	Accession numbers SRR10382244 - SRR10382249 under the NCBI BioProject PRJNA586863
HG002	Illumina reads	ftp://ftp-trace.ncbi.nlm.nih.gov/giab/ftp//data/AshkenazimTrio/HG002_NA24385_son/NIST_HiSeq_HG002_Homogeneity-10953946/NHGRI_Illumina300X_AJtrio_novoalign_bams/HG002.hs37d5.60x.1.bam
HG002	Ground truth	https://ftp-trace.ncbi.nlm.nih.gov/giab/ftp/release/AshkenazimTrio/HG002_NA24385_son/NISTv3.3.2/GRCh37/
HG005	CLR reads	https://ftp-trace.ncbi.nlm.nih.gov/giab/ftp/data/ChineseTrio/HG005_NA24631_son/MtSinai_PacBio/PacBio_minimap2_bam/
HG005	ONT reads	https://s3-us-west-2.amazonaws.com/human-pangenomics/index.html?prefix=NHGRI_UCSC_panel/HG005/nanopore/Guppy_4.2.2/01_09_20_R941_GM24631_*_Guppy_4.2.2_prom.fastq.gz
HG005	Ground truth	ftp://ftp-trace.ncbi.nlm.nih.gov/giab/ftp/release/ChineseTrio//HG005_NA24631_son/latest/GRCh37/supplementaryFiles/
HG01109	CLR reads	https://s3-us-west-2.amazonaws.com/human-pangenomics/NHGRI_UCSC_panel/HG01109/PacBio_CLR/
HG01109 mother	Illumina reads	https://s3-us-west-2.amazonaws.com/human-pangenomics/NHGRI_UCSC_panel/HG01108/illumina/
HG01109 father	Illumina reads	https://s3-us-west-2.amazonaws.com/human-pangenomics/NHGRI_UCSC_panel/HG01107/illumina/
A.thaliana F1	CLR reads	Accession numbers SRR3405291 - SRR3405326 under the NCBI BioProject PRJNA314706
A.thaliana CVI-0	CLR reads	Accession numbers SRR3405327-SRR3405386 under the NCBI BioProject PRJNA314706
